# Responses of ecosystem water use efficiency to spring snow and summer water addition with or without nitrogen addition in a temperate steppe

**DOI:** 10.1371/journal.pone.0194198

**Published:** 2018-03-12

**Authors:** Xiaolin Zhang, Penghui Zhai, Jianhui Huang, Xiang Zhao, Kuanhu Dong

**Affiliations:** 1 College of Animal Science and Veterinary Medicine, Shanxi Agricultural University, Taigu, China; 2 State Key Laboratory of Grassland Agro-ecosystems, Lanzhou University, Lanzhou, China; 3 College of Agriculture, Shanxi Agricultural University, Taigu, China; 4 State Key Laboratory of Vegetation and Environmental Change, Institute of Botany, the Chinese Academy of Sciences, Beijing, China; Tennessee State University, UNITED STATES

## Abstract

Water use efficiency (WUE) is an important indicator of ecosystem functioning but how ecosystem WUE responds to climate change including precipitation and nitrogen (N) deposition increases is still unknown. To investigate such responses, an experiment with a randomized block design with water (spring snowfall or summer water addition) and nitrogen addition was conducted in a temperate steppe of northern China. We investigated net ecosystem CO_2_ production (NEP), gross ecosystem production (GEP) and evapotranspiration (ET) to calculate ecosystem WUE (WUEnep = NEP/ET or WUEgep = GEP/ET) under spring snow and summer water addition with or without N addition from 2011 to 2013. The results showed that spring snow addition only had significant effect on ecosystem WUE in 2013 and summer water addition showed positive effect on ecosystem WUE in 2011 and 2013, as their effects on NEP and GEP is stronger than ET. N addition increased ecosystem WUE in 2012 and 2013 both in spring snow addition and summer water addition for its increasing effects on NEP and GEP but no effect on ET. Summer water addition had less but N addition had greater increasing effects on ecosystem WUE as natural precipitation increase indicating that natural precipitation regulates ecosystem WUE responses to water and N addition. Moreover, WUE was tightly related with atmospheric vapor-pressure deficit (VPD), photosynthetic active radiation (PAR), precipitation and soil moisture indicating the regulation of climate drivers on ecosystem WUE. In addition, it also was affected by aboveground net primary production (ANPP). The study suggests that ecosystem WUE responses to water and N addition is determined by the change in carbon process rather than that in water process, which are regulated by climate change in the temperate steppe of northern China.

## Introduction

In terrestrial regions, water is the most important factor limiting plant growth and ecosystem processes, so plants have evolved various adaptive water-related strategies to survive, such as reducing water loss and increasing water absorption [1]. Therefore, it is a complex question to determine how plant and ecosystem respond to water availability [2], especially in the face of global climate change, including changes in precipitation patterns and increases in nitrogen (N) deposition. As plant carbon cycle processes following with water use like transpiration, so the balance between them are the key points to study ecosystem responses to global climate change, which remain gaps in global change studies [3, 4].

Ecosystem water use efficiency (WUE) is an effective tool for assessing ecosystem responses to global climate change linking carbon and water cycles [5]. It is calculated by the rate of carbon uptake per unit of water lost, liking the ratio of net ecosystem CO_2_ production (NEP) to evapotranspiration (ET) [6–8] or gross ecosystem photosynthesis (GEP) to evapotranspiration (ET) [9–11]. Previous studies showed that ecosystem WUE based on NEP is indicator of the ecosystem carbon cycle giving their associations with both carbon assimilation (GEP) and carbon release (ecosystem respiration) processes that response different inherently to change in water loss [12, 13]. In addition, ET was also reported to be an important index for evaluating ecosystem water flux integrating by soil evaporation and plant transpiration [14]. Therefore, WUE is useful to make projections about potential changes in project global carbon and water cycles, which control the responses of ecosystem processes to global climate change.

Precipitation is expecting to increase by about 30% of mean annual precipitation at the last three decades of the 21st century in North China [15], so it will influence ecosystem carbon and water processes inevitably. Many studies have focused on the responses of WUE to increasing precipitation, most of which showed that WUE would decrease [16–18], but others instead showed that WUE would increase [11, 19] or remain unchanged [20]. In addition, some studies found that the responses of WUE depended on natural precipitation [3, 21–23]. There were no consistent conclusions on ecosystem WUE responses to precipitation indicating more manipulative experiments needed to be carried out. Snowfall is also an important form of precipitation, the depth of which has shown a tendency to increase in northern Eurasia during the past half-century [24] and the prediction in northern China showed that the amount of snowfall will increase greatly in the near future [25]. Snowmelt promotes soil moisture which is in favor of plant growth [26] but melting of snow can also take more nutrients away stored in the snowpack before plants need them [27, 28]. Supplement snowfall stimulates NEP and GEP simultaneously in the mixed prairie [29, 30], but has no effect on ecosystem respiration in an alpine bog [31]. However, few studies have focused on the effect of snowfall on WUE in recent years.

As the different responses of NEP, GEP and ET to N addition, so evaluating their relative contributions to ecosystem WUE is important to understand the impacts of N addition on ecosystem carbon and water cycles. Some studies showed N addition increased WUE by increasing carbon fluxes, while leaving ET unchanged [21, 32], but others found no obvious difference [2, 33]. However, the information on how ecosystem WUE responds to N addition remains poorly understood until now. In addition, a previous study found that the effect of N addition on water use efficiency was regulated by precipitation during the growing seasons in arid and semi-arid areas [32]. Thus, the effect of N addition on ecosystem WUE may also be regulated by precipitation in our study site and with water added simultaneously may have potential interaction. Unfortunately, how precipitation influences the response of ecosystem WUE to N addition is still lacking.

The temperate steppe in Inner Mongolia, northern China, characterized with arid or semiarid grassland which is sensitive to climate change, including increases in precipitation and N deposition [34, 35]. To estimate ecosystem carbon cycle and water cycle processes to global climate changes, a field study was conducted to investigate ecosystem WUE responses to water addition (spring snow addition and summer water addition) and N addition in three consecutive years. In this study, we attempt to answer the following questions: (1) How do WUE and its components respond to spring snow and summer water addition? (2) How do WUE and its components respond to N addition? We also (3) explore what is the main factor driving the inter-annual variations of water addition and N addition on WUE.

## Materials and methods

### Site description

The field experiment site is located at Inner Mongolia Grassland Ecosystem Research Station, Institute of Botany, the Chinese Academy of Sciences, which is in a region of semiarid temperate steppe. The community is dominated by C_3_ grasses, such as *Stipa grandis* and *Leymus chinensis*. The long-term (1982–2013) mean annual temperature is 0.4 °C and annual precipitation is about 333.3 mm, 95.4% of which falls between November and the following year September, with only 4.6% falling in October. Data of rainfall is measured from an adjacent eddy flux tower, and snowfall amount is getting manually and describes as water equivalent of snow.

### Experimental design and treatments

In the study treatments, a randomized block design was applied with two levels of water addition, namely, either spring snow addition (0, 25 mm) or summer water addition (0, 100 mm), and N addition (0, 10 g N m^−2^ a^−1^). There were 30 plots including six treatments multiplied by five replicates. Each plot had an area of 25 m^2^ (5 m × 5 m) and a distance of at least 1 m between two adjacent plots. The six treatments were as follows: control (N0W0), spring snow addition (N0W1), summer water addition (N0W2), nitrogen addition (N1W0), spring snow with nitrogen addition (N1W1), and summer water with nitrogen addition (N1W2). Plots with spring snow addition were supplied with snow equivalent to 25 mm of water at the beginning of March, as the snow melted away after middle of March [12]. While plots with summer water addition (total 100 mm) were treated with 10 mm of water weekly from June 15 every year by a total of 10 times, from 2010 to 2013. Nitrogen addition treatment involved the supply of urea in early July from 2009 to 2013.

### Water use efficiency (WUE)

Ecosystem carbon exchange and water exchange were measured by a transparent chamber (0.5 × 0.5 × 0.5 m^3)^ with two small fans for mixing air attached to an infrared gas analyzer (IRGA, LI-840; LI-COR Inc., Lincoln, NE, USA). The chamber was placed on a stainless-steel frame (0.5 × 0.5 m^2^), which was inserted into the soil to a depth about 8 cm, with about 2 cm left aboveground. Measurements were performed during 80 s at 1-s intervals, which were repeated three times every month in 2011, 2012 and 2013 during the growing season from May to September. Water, CO_2_ concentration, cell temperature, cell pressure, and various other indicators were recorded in the course of the experiment to NEP, GEP, and ET. For more details on calculating NEP and GEP, see the previous report by Zhang et al. [12]. WUE was calculated as follows: WUEnep = NEP/ET or WUEgep = GEP/ET.

### Atmospheric vapor-pressure deficit, photosynthetic active radiation, soil moisture, and aboveground net primary production

Data on atmospheric vapor-pressure deficit (VPD) and photosynthetic active radiation (PAR) were obtained from an eddy covariance tower in the vicinity (about 200 m away). Soil moisture was determined at a depth of 10 cm with a TDR-200 probe when WUE was measured.

All plants were harvested by strip clipping (1 × 0.2 m^2^) on August 9, 2010, August 7, 2011, August 13, 2012, and August 8, 2013. The samples were taken back to the laboratory, oven-dried at 65 °C to a constant weight, and the sum of plants constant values was used to represent the current year aboveground net primary production (ANPP). The values of ANPP (g m^-2^) were calculated from the sum of plants constant values divided by the strip area (0.2 m^2^).

### Statistical analysis

A linear mixed model was used to analyze the major and interactive effects of spring snow addition with N addition and summer water addition with N addition on ecosystem WUE (including WUEnep and WUEgep), ET, and soil moisture during growing seasons in 2011, 2012, and 2013. In this model, spring snow, summer water and N addition were treated as fixed factors, measuring times were treated as a repeated factor, and 30 plots were treated as a random factor. In addition, linear or nonlinear regression analysis was employed to explore the relationships of WUE with VPD, PAR, soil moisture, precipitation, and ANPP in the growing seasons. All statistical analyses were performed using SPSS 21.0 for windows (SPSS Inc., Chicago, IL, USA).

## Results

### Inter-annual variation in microclimate

**Fig 1:**
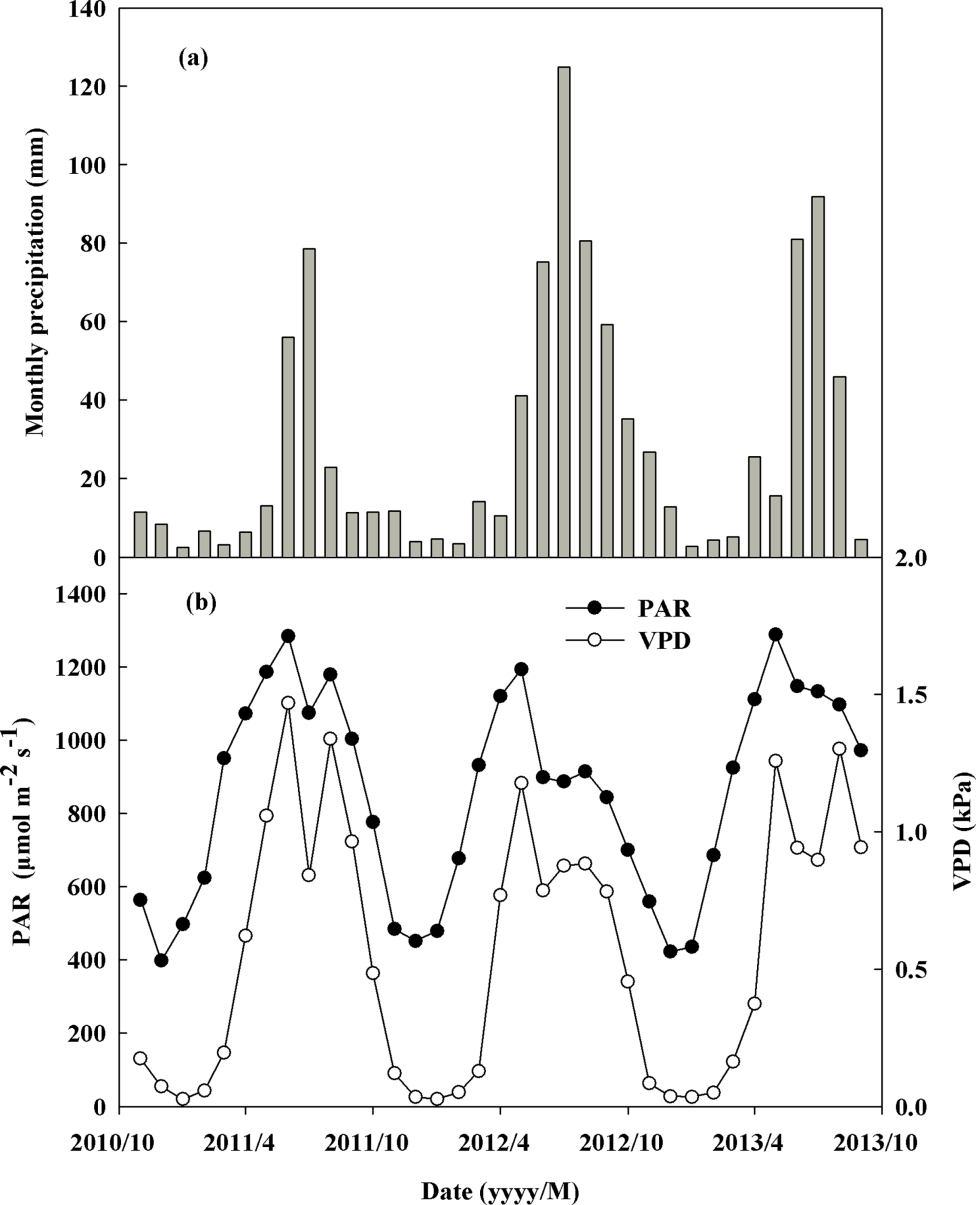
**Monthly total precipitation (mm) (A), monthly mean photosynthetic active radiation (PAR, μmol m^-2^ s^-1^) and atmospheric vapor-pressure deficit (VPD, kPa) during 8:30–10:30 from November 2010 to September in 2013 (B).** Above variables’ values are from an adjacent eddy flux tower.

The precipitation amount varied across the three years, reaching totals of 220.2 mm, 429.4 mm, and 318.9 mm from November to the following year September in 2011, 2012, and 2013, respectively (Fig 1A). In addition, the highest precipitation amount month always occurred in July of each year.

PAR also varied across the different years with its mean monthly values ranging from 451.7 to 1284.2 μmol m^−2^ s^−1^ in 2011, 422.9 to 1193.6 μmol m^−2^ s^−1^ in 2012, and 435.7 to1288.2 μmol m^−2^ s^−1^ in 2013 (Fig 1B). The mean PAR values during growing seasons were 1338.8 μmol m^−2^ s^−1^ in 2011, 1041.3 μmol m^−2^ s^−1^ in 2012, and 1277.0 μmol m^−2^ s^−1^ in 2013 (Fig 1B).

In addition, atmospheric VPD varied across the different years, with mean monthly values ranging from 0.03 to 1.47 kPa in 2011, 0.03 to 1.18 kPa in 2012, and 0.03 to 1.30 kPa in 2013, and the highest monthly mean values occurred in May or June (Fig 1B). The mean VPD values during growing seasons were 1.35 kPa in 2011, 1.08 kPa in 2012, and 1.35 kPa in 2013 (Fig 1B).

The results showed that spring snow addition only increased soil moisture by 6.9% in 2011 and 6.6% in 2012, but had no effect on it in 2013 (Table 1). Summer water addition led to an enhancement in soil moisture by 35.3%, 23.8%, and 35.1% from May to September in 2011, 2012, and 2013, respectively (Table 1). More details on soil moisture values can be found in a previously published report [12].

**Table 1:** Mixed linear model analysis results (*P*-values) on effects of water addition (W) as either spring snow addition or summer water addition, nitrogen addition (N), and their interactions on ecosystem water-use efficiency (WUEnep, WUEgep), evapotranspiration (ET) and soil moisture (SM, V/V%) in 2011–2013.

Water addition	Year	Treatment	WUEnep	WUEgep	ET	SM
Spring snow	2011	W	0.09	0.12	0.22	0.04
		N	0.32	0.07	0.64	0.33
		W*N	0.09	0.38	0.46	0.91
	2012	W	0.20	0.17	0.32	<0.001
		N	0.001	0.02	0.53	<0.001
		W*N	0.61	0.54	0.43	0.007
	2013	W	0.04	0.07	0.34	0.10
		N	<0.001	0.22	0.23	<0.001
		W*N	0.61	0.81	0.75	0.56
Summer water	2011	W	0.004	002	0.003	<0.001
		N	0.47	<0.001	0.27	0.15
		W*N	0.08	0.46	0.95	0.02
	2012	W	0.84	0.50	0.55	<0.001
		N	<0.001	0.02	0.79	<0.001
		W*N	0.50	0.10	0.71	0.15
	2013	W	<0.001	0.02	0.61	<0.001
		N	0.01	0.04	0.69	<0.001
		W*N	0.09	0.27	0.53	0.51

### Effects of water addition and nitrogen addition on ecosystem WUE

**Fig 2:**
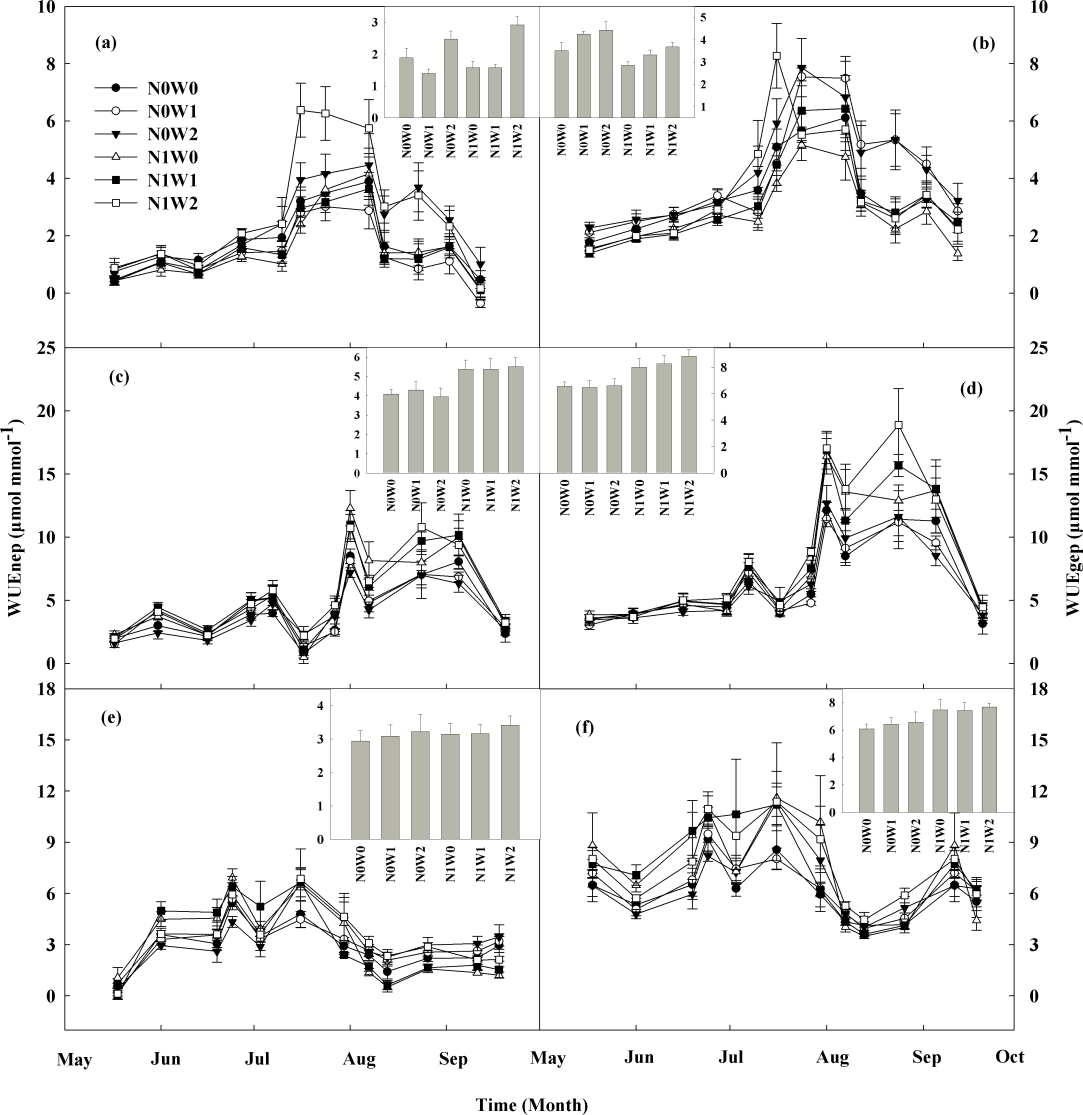
**Seasonal dynamics of water use efficiency (WUEnep and WUEgep, μmol mmol^-1^) from May to September in all treatments in 2011 (A and B), 2012 (C and D), and 2013 (E and F) respectively with averages being given in the inset figures.** Control (N0W0, solid circle), spring snow addition (N0W1, hollow circle), summer water addition (N0W2, solid triangle), nitrogen addition (N1W0, hollow triangle), spring snow with nitrogen addition (N1W1, solid square), summer water with nitrogen addition (N1W2, hollow square).

Water addition showed significant positive effects on WUE across the three growing seasons. Spring snow addition led to an increase in WUEnep by 2.7% only in 2013, but not in 2011 and 2012, and it had no effects on WUEgep in the three years (Fig 2, Table 1). Summer water addition led to an enhancement in WUEnep and WUEgep by 58.1% and by 27.6% in 2011, and by 9.1% and 5.4% in 2013, but had no effect on them in 2012 (Fig 2, Table 1).

Nitrogen addition had different effects on ecosystem WUE under spring snow addition and summer water addition. The results showed that N addition under spring snow addition treatment led to an increase in WUEnep by 25.3% and WUEgep by 27.6% in 2012, and led to an increase in WUEnep by 2.3% in 2013, but had no effect on them in the other years (Fig 2, Table 1). N addition led to an increase in WUEnep and WUEgep by 39.5% and by 33.8% in 2012, and by 5.6% and by 17.0% in 2013, but led to a decrease in WUEgep by 16.9% and had no effect on WUEnep in 2011 under summer water addition treatment (Fig 2, Table 1). There were no significant interactive effect on WUEnep and WUEgep between spring snow addition and N addition or between summer water addition and N addition (Table 1).

### Effects of water addition and nitrogen addition on net ecosystem production (NEP), gross primary photosynthesis (GEP), and evapotranspiration (ET)

**Fig 3:**
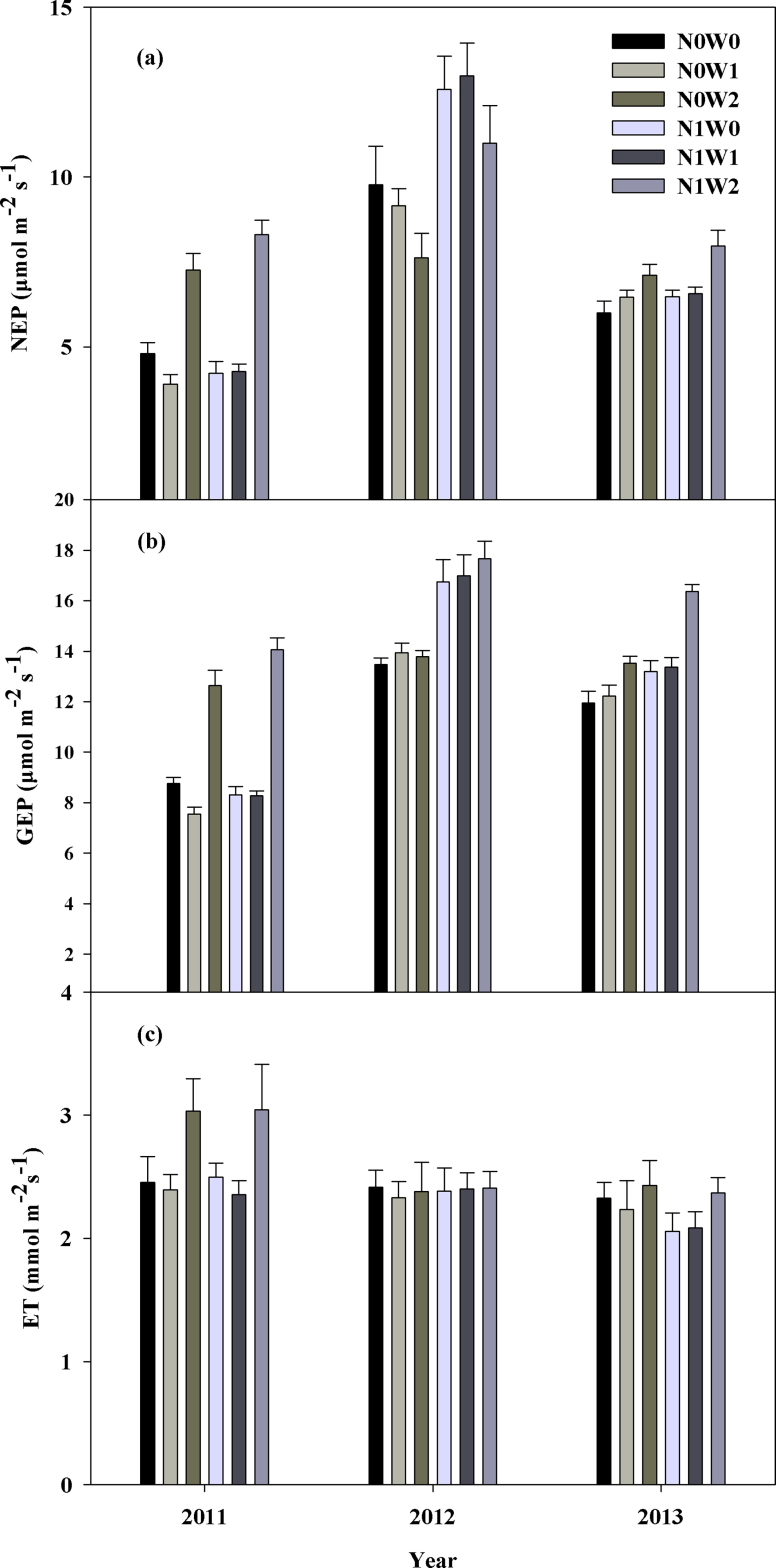
**Mean values (means ± SE) of net ecosystem production (NEP, A), gross ecosystem production (GEP, B) and evapotranspiration (ET, C) in 2011, 2012 and 2013.** Control (N0W0), spring snow addition (N0W1), summer water addition (N0W2), nitrogen addition (N1W0), spring snow with nitrogen addition (N1W1), summer water with nitrogen addition (N1W2) treatments.

Spring snow addition had no effect on NEP or GEP during the three growing seasons, with the exception that it led to a decrease in GEP by 6.0% in 2011, while summer water addition led to an enhancement in NEP and GEP by 73.8% and 47.0% in 2011, and by 20.8% and 18.7% in 2013, respectively, but led to a decrease in NEP by 17.3% in 2012 and had no effects on GEP (Fig 3A and 3B). Nitrogen addition led to an increase in NEP and GEP by 41.8% and 22.0% in 2012, and by 1.5% and 9.3% in 2013 under spring snow addition, and led to an increase in NEP and GEP by 44.2% and 28.2% in 2012, and by 12.3% and 21.0% in 2013, respectively, under summer water addition, but affected neither in 2011 (Fig 3A and 3B). More details of similar findings are presented in a previously study performed at the same site [12].

Summer water addition led to an enhancement in ET by 22.8% in 2011, but had no effects on it in the other two growing seasons. Spring snow addition had no effect on ET during all three growing seasons (Fig 3C, Table 1). However, N addition had no effect on ET during the three growing seasons, irrespective of spring snow addition and summer water addition treatments (Table 1). There were insignificant interactive effects between spring snow addition or summer water addition and N addition with regard to the influence on ET in the three years (Table 1).

### Relationships between WUE and its related factors

**Fig 4:**
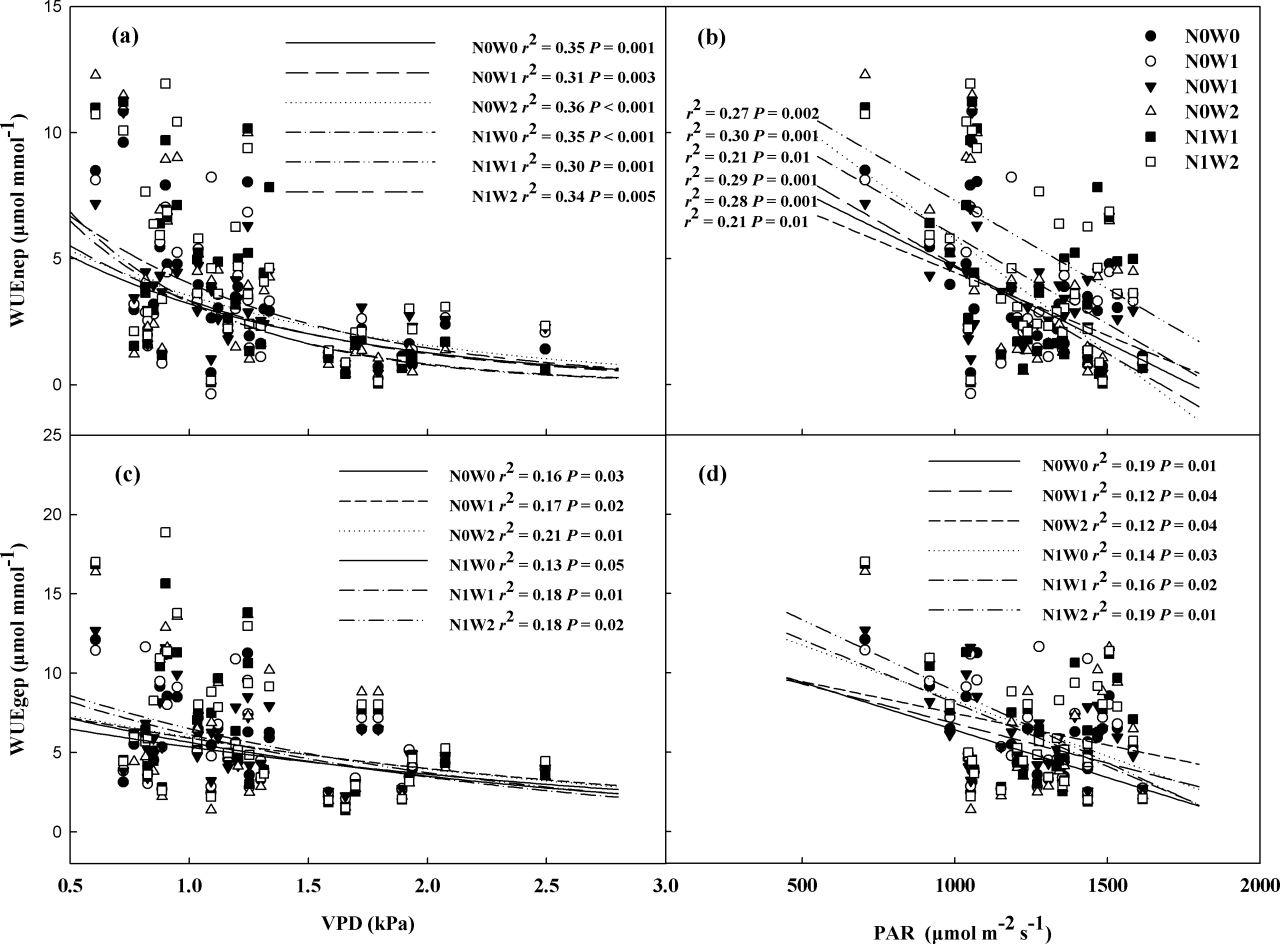
Relationships of water use efficiency (WUEnep and WUEgep, μmol mmol^-1^) with dynamics of atmospheric vapor-pressure deficit (VPD, kPa) (A and C), and photosynthetic active radiation (PAR, μmol m^-2^ s^-1^) (B and D) in all treatments across three growing seasons.

**Fig 5:**
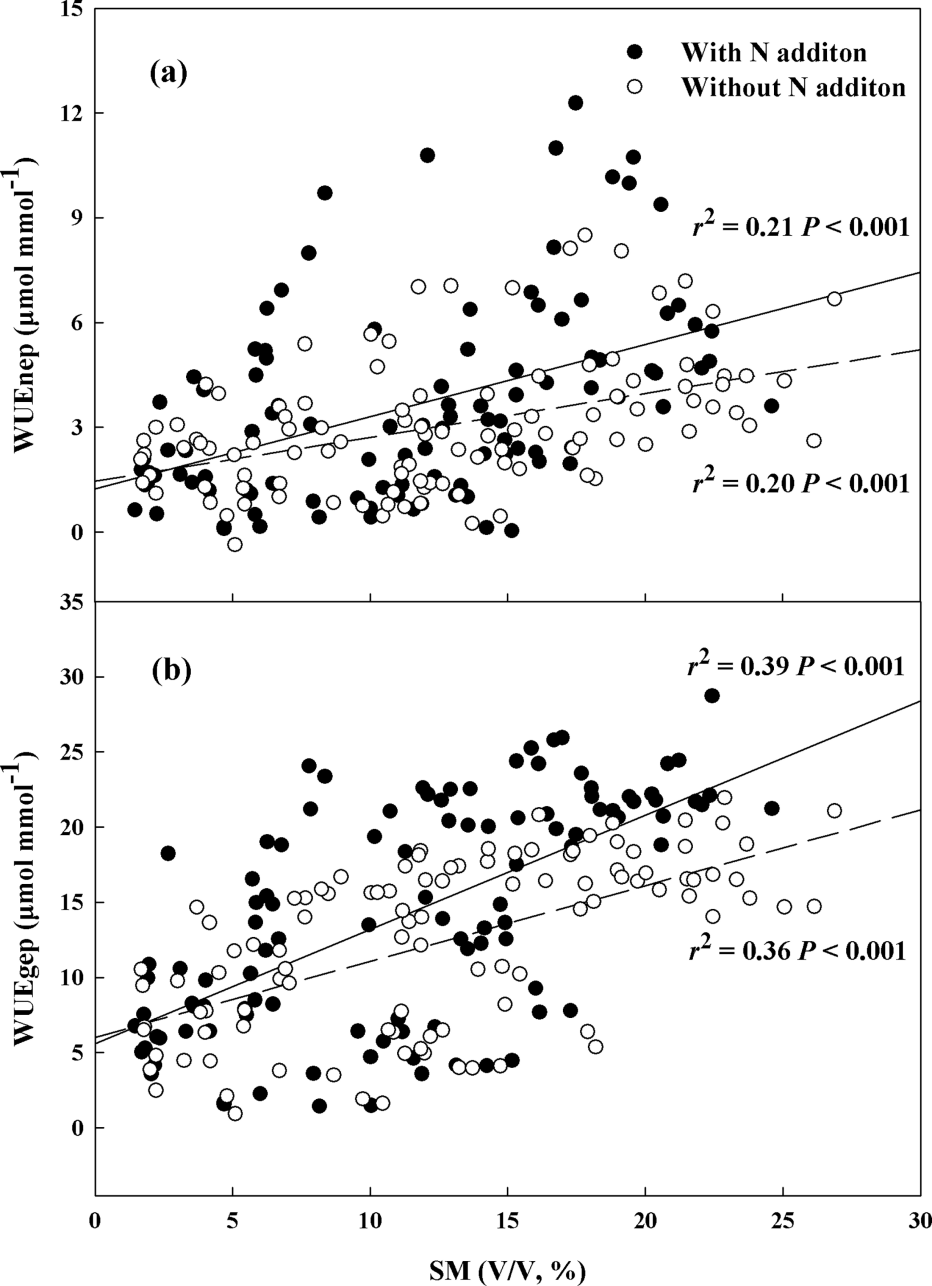
Relationships of water use efficiency (WUEnep and WUEgep, μmol mmol^-1^) with dynamics of soil moisture (SM, V/V, %) (A and B) in plots with N addition (solid symbols) and without N addition (hollow symbols) across three growing seasons.

**Fig 6:**
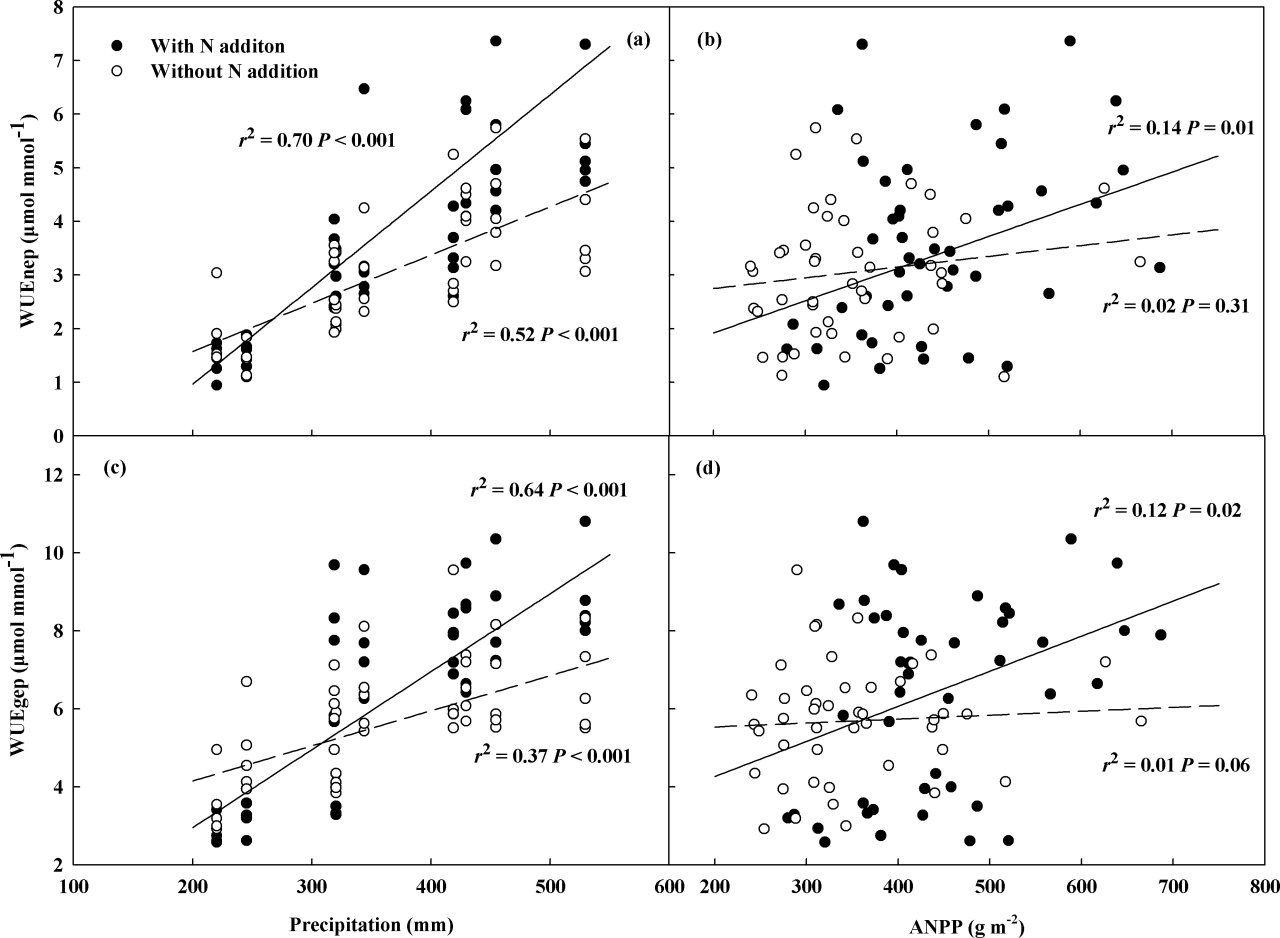
Dependence of water use efficiency (WUEnep and WUEgep, μmol mmol^-1^) on precipitation (natural rainfall + water addition) (A and C) and aboveground net primary production (ANPP) (B and D) in plots with N addition (solid symbols) and without N addition (hollow symbols) across three growing seasons.

In the study site, WUEnep and WUEgep showed negative logarithmic relationships with VPD in the six treatments during the three growing seasons (Fig 4A and 4C). In addition, WUEnep and WUEgep decreased as PAR increased in the six treatments (Fig 4B and 4D). Across the three growing seasons, WUEnep and WUEgep increased linearly with soil moisture in the water addition treatments with or without N addition, showing different sensitivity between with or without N addition (*P* = 0.01 for WUEnep, *P* < 0.001 for WUEgep) (Fig 5). The results also showed that WUEnep and WUEgep increased linearly with precipitation and ANPP upon both spring snow addition and summer water addition with or without N addition. There was significant difference between with or without N addition for precipitation (*P* = 0.01 for WUEnep, *P* = 0.03 for WUEgep) (Fig 6).

## Discussion

### Effects of water addition on ecosystem WUE

Water plays an important role on limiting the ecosystem carbon cycle and primary production, especially for semiarid regions [36], so ecosystem WUE responses to global climate change are important as an index to study water and carbon cycles [14, 37–39]. As different responses in the direction and magnitude of NEP or GEP and ET to climate change, there is uncertainty about the overall effects on WUE under various background conditions. A previous study showed that spring drought reduced WUE in a meadow steppe ecosystem by reducing GEP but increasing soil evaporation [9], suggesting a potential increase in WUE upon the addition of spring water. However, the addition of spring snow had a positive effect on WUEnep in 2013 as the increasing trend of NEP and no change of ET, via relatively low snowfall with a later thaw [40]. In addition, it showed a weak effect on WUEnep in the other two years and on WUEgep in the three years as a result of it having insignificant effects on GEP, NEP, ET, and ANPP. This indicates that the effect of snowfall addition on WUE is regulated by the amount of natural snowfall and the amount of time it takes to thaw.

The addition of summer water increased WUE more than that of spring snow in the three growing seasons. The addition of summer water had greater effects on increasing NEP and GEP than ET, resulting in positive responses of WUEnep and WUEgep. This has also been reported in a previous study performed in a similar area [11]. In the study site, summer water addition increased soil moisture to increase plant growth and it increased ANPP to increase community coverage, resulting in an increase in ecosystem carbon exchange [40]. In addition, increasing soil moisture stimulated plant transpiration following with greater photosynthesis but decreased soil evaporation by enhancing the shading effect leading to no change in ET finally [41]. Owing to the inconsistent changes in direction of carbon cycle and ET could partly explain the findings for ecosystem WUE. Ecosystem WUE, irrespective of the magnitude of responses of WUEnep or WUEgep to summer water addition, varied during the three years, with an increase in value in 2011 compared with that in 2013, but no change in 2012, suggesting that natural precipitation plays a key role in the effect of water addition on ecosystem WUE. The findings of this study suggest that the effects of summer water addition on ecosystem carbon assimilation are greater than those on water release.

### Effects of N addition on ecosystem WUE

Previous studies found that the ratios of plant transpiration to soil evaporation increased significantly with above-ground biomass and plant growth, for the increase in plant transpiration and decrease in soil evaporation resulting in no responses of ET to N addition [14, 32]. The tradeoff between plant transpiration and soil evaporation suggesting that ET maybe independent on N deposition ascribing to N stimulated plant growth. As WUE responses to N addition involved an integrated effect on NEP and GEP with ET, they will depend more on NEP and GEP than ET. This suggests that ecosystem WUE responses to N addition are determined by the change in carbon process rather than that in water process.

Nitrogen addition had different effects on WUE in both spring snow addition treatment and summer water addition treatment during the three growing seasons. The results showed that N addition increased ecosystem WUE in 2012 and 2013 under both spring snow and summer water addition, via its increasing effects on NEP and GEP, but had no effect on ET. Similar results have been reported in previous studies [11, 32]. On the other hand, the decrease in soil moisture among the three growing seasons owing to the plant growth assimilated nutrient process following with water cycle, as previous studies showed [12], indicating that soil moisture is an important index of ecosystem WUE response to N addition. Moreover, N addition showed no effect on WUEnep and caused a decrease of WUEgep in a relatively dry year, but increased them in a normal precipitation year (with precipitation close to its long-term mean value) and a relatively wet year, with the greater increase following an increase in precipitation. This suggests that the effect of N addition on ecosystem WUE depends on natural precipitation.

### Ecosystem WUE responses to climate change

Ecosystem WUEnep and WUEgep could be partly explained by VPD and PAR in all treatments during the three growing seasons, suggesting that they are important determinants of WUE. Many studies reported that VPD is a major factor controlling WUE variation [17, 19, 42, 43], because it represents atmospheric evaporative demand, which in turn affects stomatal conductance [44, 45]. At our study site, we observed that WUE logarithmically decreased with increasing VPD for VPD reduced crabon cycle but increased ET [46], which reported the similar tendency in a previous study [47]. The tight relationship of ecosystem WUE with VPD as well as soil moisture across three growing seasons suggesting that water availability plays an important role on ecosystem WUE. In addition, PAR is a variable representing the supply of energy to an ecosystem, which controls the carbon cycle directly and affects air temperature to influence the water balance between the atmosphere and the plant indirectly [46, 47]. Ecosystem WUE decreased as PAR increasing for the asynchronous response of carbon and water cycles to PAR, which found in previous studies [47, 48]. This study showed that variation of VPD and PAR could best account for changes in both WUEnep and WUEgep, indicating that they are the main drivers of the carbon and water cycles, directly or indirectly influencing ecosystem WUE.

It is inevitable that the cycling of water and carbon will exhibit responses to long-term climate change, such as changes in seasonal and inter-annual precipitation [39, 49]. Previous studies reported that WUE has a strong relationship with ecosystem structure and function, and that precipitation is the dominant factor affecting it [11, 50]. In this study, WUEnep and WUEgep increased with increasing precipitation suggesting that precipitation is a critical factor regulating WUE and it also indicated that climate variables are potential drivers of ecosystem carbon and water processes. In addition, as there was a significant difference in the response of WUE to precipitation under with or without N addition treatment indicating that N plays a positive regulatory effect on precipitation to WUE when precipitation amount is relative abundant (close to or greater than long-term mean value).

## Conclusion

A field experiment was conducted to study ecosystem WUE to evaluate carbon and water cycle responses to precipitation including spring snow or summer water addition and N deposition. The results found that water and N addition showed no effects on ET from the increase in plant transpiration and decrease in soil evaporation suggesting that they had limit effect on ecosystem water cycle. Spring snow addition showed weak effect on ecosystem WUE for its insignificant effect on GEP, NEP and ET. Summer water addition showed positive effect on WUE as the greater effects on NEP and GEP than ET with greater increase magnitude as natural precipitation decrease suggesting that natural precipitation play a key role on ecosystem WUE under water addition. N addition increased WUE both in spring snow addition and summer water addition in relative wet years but decreased ecosystem WUE in dry year suggesting that N plays a positive regulation on effect on WUE under relative abundance water background and indicating that N addition effect on WUE is depending on natural precipitation. In addition, precipitation, ANPP, soil moisture, VPD and PAR are main factors influencing WUE. In the study site, ecosystem WUE responses to water and N addition is determined by the change in carbon process rather than that in water process. The findings will facilitate to study terrestrial ecosystem carbon and water cycles process responses to climate change including precipitation and N deposition.

## Supporting information

S1 Dataset The related values of precipitation (PPT, mm), photosynthetic active radiation (PAR, μmol m-2 s-1), atmospheric vapor-pressure deficit (VPD, kPa), soil moisture (SM, V/V%) and aboveground net primary production (ANPP) in study. (XLSX)

S2 Dataset Individual data points in an.xlsx format for Figs 2 and 3. (XLSX)
